# Predictors of effective management of acute pain in children within a UK ambulance service: a cross-sectional study

**DOI:** 10.29045/14784726.2019.12.4.3.58

**Published:** 2019-12-01

**Authors:** Gregory Adam Whitley, Pippa Hemingway, Graham Richard Law, Caitlin Wilson, A. Niroshan Siriwardena

**Affiliations:** University of Lincoln: ORCID iD: https://orcid.org/0000-0003-2586-6815; University of Nottingham: ORCID iD: https://orcid.org/0000-0003-1944-8166; University of Lincoln: ORCID iD: https://orcid.org/0000-0001-7904-0264; North West Ambulance Service NHS Trust: ORCID iD: https://orcid.org/0000-0002-9854-4289; University of Lincoln: ORCID iD: https://orcid.org/0000-0003-2484-8201

## Abstract

**Introduction:**

Pre-hospital pain management in children is poor, with very few children in pain receiving analgesia. Without effective pain treatment, children may suffer long-term changes in stress hormone responses and pain perception and are at risk of developing posttraumatic stress disorder. We aimed to identify predictors of effective management of acute pain in children in the pre-hospital setting.

**Methods:**

A retrospective cross-sectional study using electronic clinical records from one large UK ambulance service between 1 October 2017 and 30 September 2018 was performed using multi-variable logistic regression. We included all children < 18 years suffering acute pain. Children with a Glasgow Coma Scale of < 15, no documented pain or without a second pain score were excluded. The outcome measure was effective pain management (abolition or reduction of pain by ≥ 2 out of 10 using the numeric pain rating scale, Wong and Baker FACES^®^ scale or Face, Legs, Activity, Crying and Consolability (FLACC) scale).

**Results:**

A total of 2312 patients were included for analysis. Median (IQR) age was 13 (9–16), 54% were male and the cause of pain was trauma in 66% of cases. Predictors of effective pain management include children who were younger (0–5 years) compared to older (12–17 years) (adjusted odds ratio (AOR) 1.57; 95% confidence interval (CI) 1.21–2.03), administered analgesia (AOR 2.35; CI 1.94–2.84), attended by a paramedic (AOR 1.39; CI 1.13–1.70) or living in an area of medium deprivation (index of multiple deprivation (IMD) 4–7) compared to children in an area of high deprivation (IMD 1–3) (AOR 1.41; CI 1.10–1.79). Child gender, type of pain, transport time and clinician experience were not significant.

**Conclusion:**

These predictors highlight disparity in effective pre-hospital management of acute pain in children. Qualitative research is needed to help explain these findings.

